# Prevalence of high-risk behaviors in reproductive age women in Alborz province in 2019 using unmatched count technique

**DOI:** 10.1186/s12905-020-01056-9

**Published:** 2020-08-31

**Authors:** Samira Bahadivand, Amin Doosti-Irani, Manoochehr Karami, Mostafa Qorbani, Younes Mohammadi

**Affiliations:** 1 Department of Epidemiology, School of Public Health, Hamadan University of Medical Sciences, Hamadan, Iran; 2 Students Research Committee, Hamadan University of Medical Sciences, Hamadan, Iran; 3 Modeling of Noncommunicable disease Research Center, School of Public Health, Hamadan University of Medical Sciences, Hamadan, Iran; 4 Research Center for Health Sciences, School of Public Health, Hamadan University of Medical Sciences, Hamadan, Iran; 5 Alborz University of Medical Sciences, Alborz, Iran; 6 Social Determinants of Health Research Center, School of Public Health, Hamadan University of Medical Sciences, Opposite of Mardom Park, Hamadan, Iran

**Keywords:** Women’s health, High risk behavior, Iran, Prevalence

## Abstract

**Background:**

Our knowledge on the prevalence of high-risk behaviors among women of Alborz is not reliable due to the sensitivity of the issue. This study aimed to estimate the prevalence of seven risk behaviors among the reproductive age women in Alborz Province, Iran.

**Method:**

In this cross-sectional study, 2000 women were randomly selected from a registered healthcare system in 2019. A researcher-made questionnaire was used to collect the required data. The prevalence of the risk behaviors including drug abuse, hookah, alcohol drinking, tobacco smoking, extramarital intercourse, and gambling was estimated using the unmatched count technique, and the prevalence rate was reported at a 95% confidence interval.

**Results:**

The response rate for this study was 95%. The estimated prevalence for drug abuse, hookah, alcohol drinking, tobacco smoking, extramarital intercourse, and gambling were 3% (95%CI: 2.78 to 3.22), 10.5% (95%CI: 10.29 to 10.71), 7% (95%CI: 6.78 to 7.22), 10% (95%CI: 9.78 to 10.22), 8.7% (95%CI, 8.29 to 8.71), and 7.5% (95%CI, 4.71 to 7.28), respectively.

**Conclusion:**

High risk behaviors are highly prevalent among Iranian women. Enforcing laws, prohibition, marketing restrictions, increasing taxation, expanding treatment, promoting condom distribution and providing community-based service are recommended to reduce the effects of high risk behaviors among women.

## Background

High-risk behaviors are the factors that increase the probability of adverse health outcomes include communicable and non-communicable diseases [1]. These behaviors mainly include tobacco and hookah smoking, alcohol drinking, drug abuse, sexual contact, gambling, which impose many risks on the health systems [2]. Due to the growing trend of such behaviors, they are being continuously monitored by health organizations [3, 4].

Tobacco use is one of the major determinants of increasing in burden of diseases in the world, especially chronic diseases including cardiovascular diseases, respiratory diseases, cancers, and strokes. Out of 1.1 billion smokers in the world, 80% live in low- and middle-income countries. According to available statistics, 11.9% of Iranian people aged over 15 years are smokers [5, 6]. In recent years, hookah smoking has experienced a significant rise in Iran and other Eastern Mediterranean Region countries [7, 8], with a prevalence rate of 11.3% in Iran and about 24% worldwide [6]. Consumption of illicit substances such as narcotics, psychotropic substances, and supplements is a major problem, and interest in using such substances is still increasing in many communities. Accordingly, 2.3 million addicts live in the Middle East, the Near East, and Southwest Asia. The prevalence rate of drug use has been estimated to be 2.4% in Iran [9–11]. Alcohol consumption, as another risk behavior, is associated with adverse outcomes such as addiction, depression, suicide, interpersonal problems with family and friends, driving-related injuries or death, which may negatively influence the economy of the family and society [12]. The estimated prevalence rate of alcohol consumption is less than 1% in Iran. The global prevalence rate of alcohol consumption disorders among adults has been estimated to range from 0 to 16% [13]. Any unprotected sex may cause many diseases such as HIV/AIDS, hepatitis, syphilis, gonorrhea, and other STDs transmitted by sexual contact [14]. According to a global survey, 35% of women experienced a type of physical or sexual violence [13, 15]. Another risk behavior is gambling that, nowadays, due to the development of Internet technologies, impose a huge burden on people, communities, and therefore has been announced as a public health concern [16] which cause serious health problems such as addiction and depression [17].

Women, especially in reproductive ages, are one of the vulnerable groups in the communities may be influenced by high risk behaviors. They have delicate and complex system in the body that require to take steps to protect them against communicable and non-communicable diseases and injury, and prevent health problems. High risk behaviors in women have been presented as a key mechanism for the general deterioration of health status which directly effects on family, children and finally on development of the countries. Accordingly, Women’s reproductive age health are high priorities for health provider agencies [18, 19].

Examining literature show that number of studies on prevalence of risk behaviors in women of Alborz is limited. Only information on smoking and hookah is available for women of Alborz. These studies show that prevalence of smoking and hookah in Alborz province is 13.2 and 0.3%, respectively [20]. Unfortunately, no study reported prevalence of alcohol drinking, drug abuse, sexual contact, gambling in women of Alborz province. This lack of information arise from cultural and religious limitations of Iranian community make researchers not to investigate these sensitive issues. In fact, Iranian people would not like to answer to private questions, and usually they give a wrong answer. Moreover, Alborz province is the latest one established province in 2011, which until before the establishment, was a part of Tehran province, capital of Iran.

Despite availability of knowledge on the prevalence of risk behaviors in Iranian women, the validity of these statistics is questionable. Generally, due to potential consequences such as shame and wickedness, rejection of society, fear of imprisonment, or even beating and execution (stoning) at community level, it is difficult to measure the prevalence of such behaviors correctly. One of the effective solutions to overcome such problems is to use indirect methods such as the unmatched count technique (UCT), which has two sets of questions: one with sensitive questions and the other without sensitive question. Studies have shown that estimates produced using this method are reliable and valid [21–23].

In this study, we aimed to estimate the prevalence of high risk behaviors among women residing in Alborz Province, Iran, using UCT.

## Methods

### Setting and study population

This descriptive cross-sectional study was performed in Alborz Province, central Iran.

In this study, we included women aged 15 to 49 years resided in Alborz province. We excluded the women who either did not satisfy with participation in study or had diseases such as dementia.

Based on the 1% prevalence rate of alcohol consumption, we reached the sample size of 1850 individuals [6].

### Sampling and sample size

Study Population was all women aged 15 to 49 years in Alborz province in 2019. Accordingly, our sampling frame was list of all women age 15 to 49 years reside in Alborz province which have been registered in the Iranian integrated health system (abbreviated in Persian SIB). In this system, each women has a unique code and all characteristics related with health and fertility status is recorded. We used stratified simple random sampling technique to choose the participants of the study. In this study, our strata’s were six cities of Alborz province include Karaj and Fardis, Savojbolagh, Taleghan, Nazarabad, Mahdasht and Eshtehard. Proportional to population size of each city, the required sample was extracted. Afterwards, using site random.org, we generated a series of random number to choose unique code of participants. Subsequently, by phone, we invited the selected women to attend in health center of the county, and information about objectives of research and how to fill in the questionnaire was presented. If participant disagree with attendance in health center or participation in study after attendance in health center, alternative participant randomly was chose and invited. The selected individuals randomly were allocated to one of two groups include list A or B, in such a way that each group fill in the own questionnaire. Each questionnaire include 12 questions (six sets of relevant questions and six sets of irrelevant questions). Order and combination of questions in each group were different. The participants are asked for completing the questionnaire as self-administered, however, if participant was illiterate, the statements were quoted, and then asked the participant for specifying number of statements are positive for them.

### Data collection tool

In this study, we used the research-made and anonymous questionnaires to collect the required data that is provided as Additional File 1. In each questionnaire, six collections of questions contained sensitive statement, while six other collection of questions did not contain sensitive statement. This questionnaires asked about the number of positive answers to a collection of questions. The participants must answer the question that “how many statements are positive for you? For example, consider the following collection:

1. I have a car
2. Last digit of my mobile phone is odd.
3. During 2018, I smoked more than 100 cigarettes
4. I am fan of Persepolis football team.
5. I prefer sour taste over sweet.

For this collection, the participant had six choices to answer: zero, one, two, three, four, and five. Therefore, they did not directly answer to the single statement, rather than specify the number of statements are positive for them. One the other hand, for other group, there was the same question but without sensitive statement (in this example, statement 3) to answer. The questionnaire of each group had six collections of questions with sensitive statement and six collections of questions without sensitive statement.

Psychometric properties of the questionnaire were assessed before study. To assure reliability of questionnaire, we did a test re-test with a two week interval. Subsequently, we calculated Intraclass Correlation Coefficient (ICC) for this test re-test. ICC ranged from 0.64 (95% CI: 0.61 to 0.70) to 0.96 (95% CI: 0.93 to 0.98). To increase validity of the questions, we used standard definitions usually presented by World Health Organization (WHO). Moreover, clarity of questions were evaluated by five experts in psychiatry and epidemiology fields, which asked them to score to each question in terms of degree of clarity. Accordingly, rate of clarity for questions were 80%. Finally, the questionnaire were reviewed by 15 participants (which selected non-randomly) to assure the questions are clearly understandable.

The seven sensitive questions used in this study were as follows:

1. During 2018, I used illicit drugs (heroin, cocaine, morphine, opium, salt, burnt, crack, LSD, grass, tramadol, hashish, and marijuana) for non-pharmaceutical purposes.
2. In the last month, I used hookah at least once.
3. In the last month, I drank alcohol at least once.
4. During 2018, I smoked more than 100 cigarettes.
5. During 2018, I had sexual intercourse with someone other than my husband.
6. During 2018, I participated in gambling activities.

### Statistical analysis

To estimate prevalence of a specified risk behavior, we calculated mean score of each collection of statement for two groups separately (with and without sensitive statement). Afterwards, we subtracted mean score of group with sensitive statement from mean score of group without sensitive statement. Therefore, this difference yielded prevalence of the specified risk factor. To estimate the variance of the prevalence to calculate confidence intervals, we summed the variances of the lists A and B in the both groups, and then subtracted them from their covariance, and divided by the sample size of study. The square root of the calculated variance divided by the sample size was used as the standard error to determine 95% confidence intervals [24].

### Ethical consideration

The study was approved by the Ethics Committee of the Hamadan University of Medical Sciences (IR.UMSHA.REC.1397.745). Additionally, the researchers stated objectives, methods and privacy and confidentiality of study for the participants clearly. Finally, as evident in first page of the questionnaire (has been provided as additional file 1), a written informed consent was obtained from participants before filling out the questionnaire. This form include two parts: information sheet and the consent certificate. This consent is documented by the signature of the participant. To increase privacy and confidentiality of participants, we did not include identifying information such as age, education level, job and residence.

## Results

Out of 2000 women 15 to 49 years invited to the study, 1894 people agree with participation in study, therefore, the participation rate for this study was 95%.

In this study, to increase confidentiality and anonymity of participants, we decided not to include demographic information such as age, education, job, etc., therefore, no demographic information is presented here.

Table 1 provides the prevalence rate of the seven high risk behaviors. As shown in the table, the highest rate belonged to hookah smoking with the prevalence rate of 10.5%, followed by cigarette smoking with the prevalence rate of 10%. Extramarital intercourse was next in rank with the prevalence rate of 8.7% compared to the remaining four risk behaviors. Similarly, 8.7% of women in the province were estimated to participate in gambling activities during the last year. Alcohol drinking with the prevalence rate of 7% was in the fifth rank among the seven risk behaviors. Moreover, the prevalence rate of illicit drugs was 3% among women in the province, which was the lowest prevalence rate among the seven risk behaviors.

**Table 1:** Prevalence of high-risk behaviors among women aged 15 to 49 years

risk behavior	Prevalence (%)	95% Confidence Interval
Drug abuse	%3	2.8 to 3.2
Hookah smoking	%10.5	10.3 to 10.7
Alcohol drinking	%7	6.8 to 7.2
Tobacco Smoking	%10	9.8 to 10.2
extramarital sex	%8.7	8.3 to 8.7
gambling	%7.5	4.7 to 7.3

## Discussion

In this study, we aimed to estimate the prevalence of a number of high risk behaviors among women in Alborz Province, Iran, with the use of UCT. The results showed that the highest rank belonged to hookah smoking with the prevalence rate of about 11%. In contrast, the lowest rank was observed to belong to drug abuse with the prevalence rate of 3%. It was also observed that all the risk behaviors indicated moderate to high prevalence rates in the women.

After reviewing the literature, we found that number of studies investigating prevalence of risk behaviors of interest in Alborz is severely limited. As mentioned in introduction, Alborz is a recently established province, which in previous was a part of Tehran provinces, therefore, information for this province is not available. However, based on results of STEPs survey, prevalence of smoking and hookah among women of Alborz was 13.2 and 0.3% [20]. There is a significant difference between our result and STEPs survey. We can mention several justifications for this difference. In fact, two studies differ in terms of how to questioning, definition of smoking and hookah, sample size, sampling technique and age structure of participants.

Give that we have not appropriate study for Alborz and to evaluate status of the province, we would use studies performed in Iran or other provinces. However, one of major difference between studies arise from different settings.

Comparing results of our study with results of other studies, we found that our results were significantly higher than those of other studies. The dominant factor affecting this difference can be the method used in the present study. A majority of studies used direct methods, which made participants unable to answer sensitive questions in a stress-free manner.

The study of Ataei on women showed that the prevalence rate of cigarette smoking, drug abuse, extramarital intercourse, and alcohol drinking was 3.31, 1.30, 8.17, and 8.17%, respectively [25]. The study used direct questions to measure such behaviors.

Another study by Taghizadeh et al. showed that 59% of women had substance abuse while 1.1% had drug injection addiction. Their study was conducted among high risk women, while we conducted our study on a general population [26].

In study by Maghsoudi performed among female college students, the prevalence rate of smoking, hookah consumption, opium consumption, alcohol consumption, and extramarital intercourse was 11.32, 15.7, 1.88, 11.9, and 13.1%, respectively. As observed, there is an inconsistency between our result and Maghsoudi’s study result. The difference may arise from variance in age and education status of the participants of two studies. Our study population was general population from aged 15 to 49 years, while age structure of Maghsoudi’s study was younger, less than 25 years. Moreover, in our study, all women regardless of education level were recruited, while in Maghsoudi’s study, participants were girls and women with academic education [27].

In a study conducted by Mohtasham Amiri (2011), the prevalence rate of alcohol consumption was 7.7% in women, which was close to the rate in our study. The prevalence rate of alcohol consumption is less than 1% in Iran. However, we obtained a higher rate in our study, which might be due to the unwillingness of many people to directly report their intake, resulting in a lower estimate [28].

Yet in another study conducted by Hessami (2017), the prevalence rate of hookah smoking was 11.3% in women, which was close to the rate in our study [29].

According to Hashemi’s study, the prevalence rate of extramarital intercourse was 2%, which was lower than the rate in our study. This is because extramarital intercourse is a social stigma in the Iranian society, and even, in global communities. As a result, people do not usually report their previous experiences correctly while using a direct method [30].

In Iran, gambling is prohibited for religious reasons. There exists no research on gambling in Iran, and thus, its prevalence rate is unavailable. In our study, the prevalence rate of gambling was 7.7%. Global statistics have shown that approximately 25% of Americans aged over 21 years have gambled in casinos in the past 12 months [31].

This study provide a reliable and clear picture of provenance of high risk behaviors among Iranian women, especially that for first time we used an indirect method in general population to obtain a statistics of interest. These results may be utilized by Iranian policy makers to better planning in health scope of women. Another significant advantage of our study was the sampling technique. We randomly selected samples from all parts of the province to cover almost the entire province. Furthermore, the sample size in our study was sufficiently large, and also, a large number of individuals participated in the study (95% of the participants). In addition, we examined the prevalence rate of gambling for the first time, and no previous study reported any results on gambling status in Iran.

On the other hand, our study had some limitations. To increase the privacy of the participants, we did not collect demographic and identifying information such as age, education, location of residence (rural/urban), etc. Therefore, we could not estimate the prevalence rate of the factors using demographic variables. Moreover, 5% of the subjects disagreed to participate in the study. This issue may affect the reliability and, to a very limited extent, validity of the results.

Improvement of knowledge of the society causes the control and prevention of high-risk behaviors, so studies should be designed to moderate and correct the risk factors and strengthen the protective factors.

To increase the privacy of subjects, and due to the high validity and reliability of indirect methods, especially UCT, such methods are suggested to be used in future studies.

## Conclusion

High risk behaviors are highly prevalent among Iranian women. Enforcing laws, prohibition, marketing restrictions, increasing taxation, expanding treatment, promoting condom distribution and providing community-based service are recommended to reduce the effects of high risk behaviors among women.

## Supplementary information

**Additional file 1.**

## Data Availability

The corresponding author is responsible for data. Access to all relevant raw data will be free to any scientist.

## Abbreviations

UCT: Unmatched Count Technique
ICC: Intraclass Correlation Coefficient
WHO: World Health Orginzation

## References

[1] Peltzer K, Pengpid S, Yung TKC, Aounallah-Skhiri H, Rehman R (2016). Comparison of health risk behavior, awareness, and health benefit beliefs of health science and non-health science students: an international study. Nurs Health Sci.

[2] Pandiyan K, Aswath M (2018). Study of prevalence of high-risk sexual behaviour and associated psychiatric comorbidity among female sex WORKERS. J Evol Med Dental Sci.

[3] Azhar A, Alsayed N (2012). Prevalence of smoking among female medical students in Saudai Arabia. Asian Pac J Cancer Prev.

[4] Poorolajal J, Mohammadi Y, Soltanian AR, Ahmadpoor J (2019). The top six risky behaviors among Iranian university students: a national survey. J Public Health.

[5] Karimy M, Niknami S, Heidarnia AR, Hajizadeh I, Montazeri A (2013). Prevalence and determinants of male Adolescents' smoking in Iran: an explanation based on the theory of planned behavior. Iran Red Crescent Med J.

[6] Hamrah MS, Harun-Or-Rashid M, Hirosawa T, Sakamoto J, Hashemi H, Emamian MH, Shariati M, Fotouhi A (2013). Smoking and associated factors among the population aged 40-64 in Shahroud, Iran. Asian Pac J Cancer Prev.

[7] Islami F, Pourshams A, Vedanthan R, Poustchi H, Kamangar F, Golozar A, Etemadi A, Khademi H, Freedman ND, Merat S (2013). Smoking water-pipe, chewing nass and prevalence of heart disease: a cross-sectional analysis of baseline data from the Golestan cohort study, Iran. Heart.

[8] Bashirian S, Barati M, Mohammadi Y, Mostafaei H (2016). Factors associated with hookah use among male high school students: the role of demographic characteristics and hookah user and non-user prototypes. J Res Health Sci.

[9] Kessler RC, Angermeyer M, Anthony JC, De Graaf R, Demyttenaere K, Gasquet I, De Girolamo G, Gluzman S, Gureje O, Haro JM (2007). Lifetime prevalence and age-of-onset distributions of mental disorders in the World Health Organization's world mental health survey initiative. World Psychiatry.

[10] Heydari ST, Izedi S, Sarikhani Y, Kalani N, Akbary A, Miri A, Mahmoodi M, Akbari M. The Prevalence of Substance use and Associated Risk Factors Among University Students in the City of Jahrom, Southern Iran. Int J High Risk Behav Addict. 2015;4(2):e22381. 10.5812/ijhrba.4(2)2015.22381. PMID: 26097836; PMCID: PMC4464575. 10.5812/ijhrba.4(2)2015.22381 PMC4464575 26097836

[11] Djalalinia S, Moghaddam SS, Peykari N, Kasaeian A, Sheidaei A, Mansouri A, Mohammadi Y, Parsaeian M, Mehdipour P, Larijani B (2015). Mortality attributable to excess body mass index in Iran: implementation of the comparative risk assessment methodology. Int J Prev Med.

[12] Verma RK, Saggurti N, Singh AK, Swain SN (2010). Alcohol and sexual risk behavior among migrant female sex Workers and male Workers in Districts with high in-migration from four high HIV prevalence states in India. AIDS Behav.

[13] Burkert NT, Rasky E, Freidl W, Grosschadl F, Muckenhuber J, Krassnig R, Gatternig R, Hofer HP (2013). Female and male victims of violence in an urban emergency room-prevalence, sociodemographic characteristics, alcohol intake, and injury patterns (vol 125, pg 134, 2013). Wien Klin Wochenschr.

[14] Jo S, Shin J, Song KJ, Kim JJ, Hwang KR, Bhally H (2011). Prevalence and correlated factors of sexually transmitted diseases-chlamydia, Neisseria, cytomegalovirus-in female rape victims. J Sexual Med.

[15] Braun S, Agolino K, Angel A, Gelos DS (2018). Prevalence of violence against women and negative predictors in female smokers attended in a smoking cessation unit in Buenos Aires city: a cross-sectional study. Tob Induc Dis.

[16] Wardle H, Reith G, Langham E, Rogers RD (2019). Gambling and public health: we need policy action to prevent harm. BMJ.

[17] Riley BJ, Larsen A, Battersby M, Harvey P (2017). Problem gambling among female prisoners: lifetime prevalence, help-seeking behaviour and association with incarceration. Int Gambl Stud.

[18] Khalid A, Khan N (2013). Pathways of women prisoners to jail in Pakistan. Health Promot Perspect.

[19] Hale DR, Viner RM (2012). Policy responses to multiple risk behaviours in adolescents. J Public Health.

[20] Varmaghani M, Sharifi F, Mehdipour P, Sheidaei A, Djalalinia S, Gohari K, Modirian M, Pazhuheian F, Peykari N, Haghshenas R (2020). Prevalence of smoking among Iranian adults: findings of the national STEPs survey 2016. Arch Iran Med.

[21] Coutts E, Jann B (2011). Sensitive questions in online surveys: experimental results for the randomized response technique (RRT) and the unmatched count technique (UCT). Sociol Methods Res.

[22] Dalton DR, Wimbush JC, Daily CM (1994). Using the unmatched count technique (UCT) to estimate base rates for sensitive behavior. Pers Psychol.

[23] Hadji M, Asghari F, Yunesian M, Kabiri P, Fotouhi A (2016). Assessing the prevalence of publication misconduct among Iranian authors using a double list experiment. Iran J Public Health.

[24] Glynn AN (2013). What can we learn with statistical truth serum? Design and analysis of the list experiment. Public Opinion Quarterly.

[25] Ataei B, Salehi M, Javadi A, KHorvash F, Mortazavi AS, Kasaeian N, Nokhodian Z, Babak A. The Frequency of high-risk behaviors in intravenous drug abusers referred to addiction prohibition centers in Isfahan, 2010. J Isfahan Med School. 2011;28(114):837–42.

[26] Taghizadeh H, Taghizadeh F, Fathi M, Reihani P, Shirdel N, et al. Drug use and high-risk sexual behaviors of women at a drop-in center in Mazandaran Province, Iran, 2014, Iran J Psychiatry Behav Sci. 2015;9(2). 10.17795/ijpbs1047. 10.17795/ijpbs1047 PMC4539583 26288640

[27] Maghsoudi A, Jalali M, Neydavoodi M, Rastad H, Hatami I, Dehghan A (2017). Estimating the prevalence of high-risk behaviors using network scale-up method in university students of Larestan in 2014. J Subst Abus.

[28] Mohtasham-Amiri Z, Jafari-Shakib A, Khalili-Moosavi A (2011). Prevalence and factors associated with ecstasy use among college undergraduates in north of Iran-2005. Asian J Psychiatry.

[29] Hessami Z, Masjedi MR, Ghahremani R, Kazempour M, Emami H (2017). Evaluation of the prevalence of waterpipe tobacco smoking and its related factors in Tehran, Islamic Republic of Iran. East Mediterr Health J.

[30] Hashemi S, Seddigh S, Tehrani FR, Khansari SMH, Khodakarami N (2013). Sexual behavior of married iranian women, attending taleghani public health center. J Reprod Infertil.

[31] Peters EN, Nordeck C, Zanetti G, O'Grady KE, Serpelloni G, Rimondo C, Blanco C, Welsh C, Schwartz RP (2015). Relationship of gambling with tobacco, alcohol, and illicit drug use among adolescents in the USA: review of the literature 2000–2014. Am J Addict.

