# Supplementary material for: Prevalence of high-risk behaviors in reproductive age women in Alborz province in 2019 using unmatched count technique
Source: BMC Womens Health. 2020 Aug 31;20:186. doi: 10.1186/s12905-020-01056-9 (PMC7461357; doi:10.1186/s12905-020-01056-9)
Supplement: Supplementary file 1 — Additional file 1. [file 12905_2020_1056_MOESM1_ESM.doc]

Dear Participant,

Greetings,

I am Samira Bhadivand, M.Sc. student in epidemiology and working on my thesis entitled”Estimating the prevalence of risk behaviors in the reproductive age women of Alborz Province”, which is approved by Hamadan university of Medical Sciences (IR.UMSHA.REC.1397.745". This questionnaire has 12 collections of the statements. In this questionnaire, you don’t choose any single statement directly, rather than you just specify the number of statements of each collection are positive for you.

**Informed consent**

I would like take part in this research questionnaire in M.Sc. in epidemiology. I understand that the research aims to collect data for Estimating the prevalence of risk behaviors in the reproductive age women of Alborz Province. The data collected in this questionnaire will be used in a M.Sc. thesis and help understand the knowledge base of prevalence of risk behaviors in the reproductive age women of Alborz Province.

**By signing this questionnaire, I confirm that:**

I am absolutely aware of the objectives, methods and privacy and confidentiality of the study.

My participation/participation of my offspring in this project is voluntary.

I have read and understood that all data provided will be treated in strict confidence

I understand that this research has been approved by Ethics Committee of Hamadan University of Medical Sciences.

I have read and understood the explanation of the research project provided to me.

**Signature**

**Note:**

Please in each collection of statement, only specify **the numbers of statements** are positive for you. **Don’t choose** any statement directly.

**Collection 1**

- I am under 30 years old.
- I drink soda or beverage with food
- I prefer the cold weather over warm weather.
- I use the train to go to Mashhad city.
- I reside in down town.

**Please specify the number of positive statements for you.**

         0   □      1  □      2 □        3 □        4  □       5□

**Collection 2**

- When I am in leisure time, I watch TV.
- I was born in the first half of the year.
- I buy books for myself at least once a year.
- I eat fast food once a month.
- The last digit of my mobile number is odd.
- During this year, I have used drugs) heroin, cocaine, morphine, opium, juice, burnt, crack, LSD, grass, grass, tramadol, cannabis(

**Please specify the number of positive statements for you.**

         0   □      1  □      2 □        3 □        4  □       5□

**Collection 3**

- I have one child under five years old.
- Type of my health insurance is social security.
- During 24 hours, I get at least six hours of night sleep
- Every day, I spend some time on internet
- I am customer of Hamrah Aval Telecommunications Company.

**Please specify the number of positive statements for you**

         0   □      1  □      2 □        3 □        4  □       5□

**Collection 4**

- I prefer spending time with my family over cyberspace.
- Every day, I consume at least one fruit meal.
- I prefer bright hair dark hair.
- My height is less than 157 cm.
- I use the pedestrian bridge to cross the street.

**Please specify the number of positive statements for you**

         0   □      1  □      2 □        3 □        4  □       5□

**Collection 5**

- I go to the dentist once a year.
- Our postal code is even.
- I prefer rainy weather over sunny weather.
- In this year, on average, I smoked hookah for at least once.
- I prefer sneakers over high heels.
- My education level is diploma and higher.

**Please specify the number of positive statements for you**

         0   □      1  □      2 □        3 □        4  □       5□

**Collection 6**

- My house building is to the south.
- I keep flowers and plants in my house.
- I tend to eat more sour foods than sweet foods.
- I choose north of Iran for weekend.
- I prefer the Persepolis football team over the Esteghlal football team.

**Please specify the number of positive statements for you.**

         0   □      1  □      2 □        3 □        4  □       5□

**Collection 7**

- I prefer to light wearing over dark.
- The first digit of my national code is even.
- I have a daughter.
- During this year, on average, I have drunk alcohol once a month.
- I enjoy cooking.
- My apartment building has four floors and more.

**Please specify the number of positive statements for you.**

         0   □      1  □      2 □        3 □        4  □       5□

**Collection 8**

- I like traveling by plane.
- I prefer sweet foods over sour.
- My shoe size is less than 38.
- In leisure time, I study.
- I wear glasses when reading.

**Please specify the number of positive statements for you.**

         0   □      1  □      2 □        3 □        4  □       5□

**Collection 9**

- I use the TV to follow the news.
- I am interested in collecting decorative items.
- I have smoked more than 100 cigarettes this year.
- I buy clothes at the beginning of each season.
- I drink four glasses of water a day.
- I listen to music for an hour every day.

**Please specify the number of positive statements for you.**

         0   □      1  □      2 □        3 □        4  □       5□

**Collection 10**

- I wake up every morning before 8 o'clock.
- I prefer short hair to long hair.
- I love watching football.
- I prefer traditional music over pop.
- I prefer the use of the phone over the internet to communicate with my friends.

**Please specify the number of positive statements for you.**

         0   □      1  □      2 □        3 □        4  □       5□

**Collection 11**

- I am a member of a sports club.
- I go to the hairdresser once a month.
- I go around the city once a month for fun.
- Waist size is less than 40 cm.
- During this year, I satisfyingly have had sex with someone outside of marriage.
- I usually go to bed every night after 12 o'clock.

**Please specify the number of positive statements for you.**

         0   □      1  □      2 □        3 □        4  □       5□

**Collection 12**

- I prefer the warm seasons of the year to the cold seasons.
- I like to wear dark colored clothes more than light colored clothes.
- I weigh less than 67 kg.
- I have more than one sister.
- I do not like fast food.
- I have been betting money or anything of value during this year.

**Please specify the number of positive statements for you.**

         0   □      1  □      2 □        3 □        4  □       5□
